# The role of antioxidant supplement in immune system, neoplastic, and neurodegenerative disorders: a point of view for an assessment of the risk/benefit profile

**DOI:** 10.1186/1475-2891-7-29

**Published:** 2008-09-30

**Authors:** Daria Brambilla, Cesare Mancuso, Mariagrazia Rita Scuderi, Paolo Bosco, Giuseppina Cantarella, Laurence Lempereur, Giulia Di Benedetto, Salvatore Pezzino, Renato Bernardini

**Affiliations:** 1 Department of Experimental and Clinical Pharmacology, University of Catania, Catania, Italy; 2 Institute of Pharmacology, Università Cattolica del Sacro Cuore, Roma, Italy; 3 IRCSS OASI Maria SS, Troina, Italy

## Abstract

This review will discuss some issues related to the risk/benefit profile of the use of dietary antioxidants. Thus, recent progress regarding the potential benefit of dietary antioxidants in the treatment of chronic diseases with a special focus on immune system and neurodegenerative disorders will be discussed here. It is well established that reactive oxygen species (ROS) play an important role in the etiology of numerous diseases, such as atherosclerosis, diabetes and cancer. Among the physiological defense system of the cell, the relevance of antioxidant molecules, such as glutathione and vitamins is quite well established. Recently, the interest of researchers has, for example, been conveyed on antioxidant enzyme systems, such as the heme oxygenase/biliverdin reductase system, which appears modulated by dietary antioxidant molecules, including polyphenols and beta-carotene. These systems possibly counteract oxidative damage very efficiently and finally modulate the activity of oxidative phenomena occurring, for instance, during pathophysiological processes. Although evidence shows that antioxidant treatment results in cytoprotection, the potential clinical benefit deriving from both nutritional and supplemental antioxidants is still under wide debate. In this line, the inappropriate assumption of some lipophylic vitamins has been associated with increased incidence of cancer rather than with beneficial effects.

## Introduction

The term "free radicals" designates a family of compounds characterized by great reactivity due to the impaired electron in the outer orbital. To this group belong reactive oxygen species (ROS), such as superoxide anion, hydroxyl radical and hydrogen peroxide, as well as reactive nitrogen species (RNS) which include nitric oxide and peroxynitrite. Although structurally different, free radicals share similar mechanisms to harm body's cells and tissues through damage on proteins, DNA and lipids [1]. The alterations of membrane functions occurring as a consequence of phospholipid modifications represent a relevant, radical species-dependent injury, either when considering the organism as a whole, or a specific integrated function, such as the immune response [2]. The potential therapeutic applications of antioxidants in free radical-related diseases led to the hypothesis of their use to slow down or reverse, for example, symptoms associated with with neurodegenerative disorders, such as Alzheimer's disease (AD), Parkinson's disease (PD), or spongiform encephalopathies. Such effect could occur through a block of proinflammatory cytokines action and the resulting oxidative damage [3-7]. However, several clinical studies demonstrated that not only malnutrition, but also the excess of certain nutrients (e.g. iron, alpha-tocopherol, beta-carotene, ascorbic acid) may set into motion oxidation phenomena and, therefore, cell injury [8,9]. Thus, it is of relevance that prior to considering introducing antioxidant therapy into mainstream medicine, significant advances in basic cell biology, pharmacology and clinical bioanalysis will be required.

### Oxidative Stress

The body is normally under a dynamic equilibrium between free radical generation and quenching. The physiological defense systems to counteract free radicals encompass endogenous enzyme systems, such as catalase, glutathione reductase and superoxide dismutase, as well as glutathione, urate and coenzyme Q, or exogenous factors (β-carotene, vitamin C, vitamin E and selenium) [10]. All these molecules have an antioxidant effect due to their ability to transform ROS into stable and harmless compounds or by scavenging both ROS and RNS with a redox-based mechanism [10]. Very recently, a main role in the fight against oxidative stress has been assumed by enzymes such as heme oxygenase (HO) and biliverdin reductase (BVR). Heme oxygenase is a microsomal enzyme which metabolizes heme into ferrous iron, carbon monoxide and biliverdin (BV); the latter is then reduced by BVR into bilirubin (BR), a molecule endowed with strong antioxidant and antinitrosative activities [11-14]. Interestingly, all these protective factors act in a concerted way, enhancing the antioxidant defense system of the cell. When the balance between ROS/RNS and antioxidants turns in favor of the former, oxidative/nitrosative stress occurs. Although oxidative stress is associated with most diseases, routine assay methods are not nowadays available in the clinical practice. A strategy widely used to determine oxidative stress is measurement of malonyldialdehyde, F2-isoprostanes, or 8-hydroxydesoxyguanosine. Actually, these molecules are regarded as the most reliable markers available [15]. A classic example of an oxidation product apparently leading to disease, is oxidized cholesterol in low-density lipoprotein (LDL), which displays a higher atherogenic potential than native LDL, and mainly involved in the pathogenesis of atherosclerosis and coronary heart disease (CHD) [16].

At the cellular level, a large body of data clearly demonstrated that ROS, when produced in low amounts and in a controlled manner, are physiological components of the signalling generated by cytokines, growth factors and neurotrophic peptides [17-22], although they may also activate apoptotic cell death [23]. Extracellularly generated ROS can diffuse through anion channels into the cytoplasm; the resulting variation in the cell redox state leads to modulation of an array of transcription factors (eg. NF-kB, AP-1), protein kinases (e.g. AKT, JNK, p38), and receptor activated MAP kinases involved in apoptosis [17,24-26]. Moreover, the proapoptotic molecules Fas and Fas ligand (FasL) undergo positive transcriptional regulation after exposure to oxidants [27]. Interestingly, Krammer and Colleagues demonstrated that in vitro administration of vitamin E suppresses FasL mRNA expression and protects T cells of HIV-1 infected individuals from Fas mediated apoptosis [28]. Moreover, it was demonstrated that administration of combinations of vitamin E and C to cultures of human umbilical vein endothelial cells (HUVEC) treated with lipopolysaccharide could prevent apoptosis by upregulation of *Bcl-2 *[29].

### Antioxidants, The Immune System And Related Disorders

The protective function against external pathogens carried out by the immune system is by itself a source of ROS, since activated neutrophils, produce free radicals to a significant extent [30]. Moreover, during the inflammatory process, activation of phagocytes through the interaction of proinflammatory mediators, or bacterial products with specific receptors results in the assembly of the multicomponent flavoprotein NADPH oxidase which catalyzes the production of large quantities of the superoxide anion radical (O_2 _^-^) [31]. In addition to classical reactive oxygen metabolites, activated neutrophils and monocytes release the hemoprotein myeloperoxidase (MPO) into the extracellular space, where it catalyzes the oxidation of Cl^- ^by H_2_O_2 _to yield hypochlorous acid (HClO) [32]. HClO is a non-specific oxidizing and chlorinating agent that reacts rapidly with a variety of biological compounds, such as sulphydryls, polyunsatured fatty acids, DNA, pyridine nucleotides, aliphatic and aromatic aminoacids and nitrogen-containing compounds [33-35]. Moreover, apart from their direct toxic effects, neutrophil-derived oxidants may promote tissue injury indirectly by altering the protease/antiprotease equilibrium that normally exists within the intestinal interstitium. The oxidative inactivation of important protease inhibitors, coupled to the oxidant-mediated activation of latent proteases, creates a favorable environment for neutrophils that allows degradation of the interstitial matrix through elastases, collagenases and gelatinases, as well as injury to epithelial cells [36,37]. However, not only immune cell produce ROS necessary for the microbicidal activity, but they are also sensitive to external ROS, due to their high polyunsaturated fatty acids (PUFA) content. Immune cells are atypical, as compared with other somatic cells, in that they contain high levels of antioxidant vitamins, presumably providing protection against lipid peroxidation and immunosuppression, both of which are well known risks posed by high PUFA content [38]. The reactivity of immune cells to exogenous ROS has been shown to be age-dependent. In fact, lymphocytes from elderly individuals appear to be more sensitive to exposure to hydrogen peroxide than those from young adults [39]. Moreover, it has been demonstrated that a micronutrient deficiency can be the cause of suppression of immune function affecting both innate T-cell-mediated immune response and adaptive antibody response, thus altering the balanced host response. Therefore, an adequate intake of vitamins and antioxidant elements seems to be essential for an efficient function of the immune system. Micronutrient deficiency occurs in various conditions, such as eating disorders, tobacco smokers, chronic diseases, aging. During aging, changes in the immune system are frequent and associated with increased susceptibility to infections. Antioxidant vitamins and trace elements contribute to maintain an effective immune response [40]. For example, administration of vitamin E supplement to healthy elderly patients produced an increased antibody titer to both hepatitis B and tetanus vaccine [41], thus enhancing T-cell mediated functions. In conclusion, maintaining adequate antioxidant status may provide a useful approach in attenuating cell injury and dysfunction observed in some inflammatory/autoimmune disorders [42,43].

Autoimmunity has been for decades considered the result of a breakdown in self-tolerance. At the present, it is known that autoimmunity is a physiological process [44]. This phenomenon becomes pathological when the number of autoreactive cells, and particularly the avidity of their receptors for autoantigens, increases [44]. Triggering of the disease usually depends both on the increase in immunogenicity of the target cell, which may be secondary to a viral infection (Chediak-Higashi syndrome and Griscelli syndrome by EBV), and on the individual's own capacity to recognize the autoantigens (HLA, or T cell repertoire in Familial hemophagocytic lymphohistiocytosis [FHL]) [45]. Moreover, apart from the genetic defects that may predispose to autoimmune diseases, one must take into account the environmental factors that are implicated in the development of such pathologies. Among them, an important role is played by xenobiotics such as chemicals, drugs and metals [46]. Iron, aluminum, and manganese readily cross the blood brain barrier via specific or non-specific carriers, and contribute to the nervous tissue damage [47,48]. The toxic effects of metals are mediated through free radical formation, or enzyme inhibition [49-53]. In addition, metals may act as immunosuppressants (cytostatically), or as immunoadjuvants (through non-specific activation of the immune response) [54,55]. Several mechanisms are proposed on how metals may act within the immune system to induce autoimmunity. Patients suffering from scleroderma develop autoantigens with metal-binding sites. After metal binding, free radical species are generated which fragment auto-antigens thereby exposing cryptic epitopes, which may then trigger autoimmunity [56,57]. Taken together, these findings underlie the importance of exogenous factors in the pathogenesis of autoimmunity. Nevertheless, all these elements do not appear sufficient to provoke *chronic *autoimmune diseases such as Multiple Slerosis (MS), myasthenia gravis, Insulin Dependent Diabetes Mellitus (IDDM) or Hashimoto's thyroiditis, and the passage to chronic disease is usually secondary to a defect in immunoregulation.

Several classes of regulatory T cells, such as Th2, CD25+ and natural killer (NK) T cells, are implied in autoimmune pathologies. In an animal model of a Th2-dominated autoimmune syndrome, the administration of the antioxidant N-acetyl-cysteine (NAC) induced a decrease in mast-cell expression of both IgE and IL-4 [58]. Of major interest is the discovery of the therapeutic potential of a new benzoquinone-containing product derived from wheat germ fermentation. This latter has been shown to have immune restorative properties because it affects the Th1/Th2 network by inhibiting the Th2 response [59]. Another beneficial effect of this molecule is its anti-metastatic activity, shown in various human malignancies and Jurkat leukemia cell line [60]. Intriguingly, the combined treatment with wheat germ and vitamin C profoundly inhibited metastasis formation in various tumor models of different origin (Lewis lung carcinoma, B16 melanoma and human colon carcinoma xenografts [HCR25]) [61]. On the contrary, wheat germ had no toxicity on peripheral blood leukocytes (PBLs) at doses that affected tumor cells. The crude powder extract of fermented wheat germ inhibits nucleic acid ribose synthesis primarily through the non-oxidative steps of the pentose cycle [60]. Curiously, another quinone compound, carnosic acid quinone, like wheat germ, recovers potent antioxidant activity upon standing [62].

Keeping in mind the importance of oxidative stress in the regulation/dysregulation of immune system, the use of antioxidants in such diseases has been reasonably proposed. Rheumatoid arthritis (RA) is a classic example of autoimmune disease. Joint inflammation in rheumatoid arthritis (RA) is characterized by invasion of T cells in the synovial space and proliferation of activated macrophages and fibroblasts in the synovial intima [63]. Therefore, in the rheumatic joint there is an increased activity of fibroblasts and leucocytes which produce ROS [64,65]. Very recently, antioxidants have been successfully used as adjuvant therapy in RA [66,67]. Although the results obtained with RA seemed to be very promising, the indiscriminate use of antioxidants in autoimmune disorders is not recommended. In fact, autoimmune lymphoproliferative syndrome (ALPS), MS, type 1 diabetes and multiple autoimmune syndrome, have been linked to decreased Fas functionality [68] and, as discussed previously, antioxidants may up-regulate Fas and FasL in vitro. Increasing evidence provides support that oxidative stress and apoptosis are closely related physiological phenomena and are implicated in diseases including autoimmune diseases. Therefore molecules that target both apoptosis-related signal transduction and oxidative stress, like antioxidants, are likely to result in the improvement of these pathologies.

A novel possible approach to modulate immune system thus preventing autoimmunity or transplant rejection is the activation of cytoprotective and antioxidant enzymes such as HO-1. Heme oxygenase-1, the inducible isoform of HO, is a key protein in the cell stress response and its up-regulation is a common event during pro-inflammatory conditions [11,69-72]. Recent work clearly demonstrated that regulatory T cells overexpress HO-1 and release CO under pro-oxidant conditions. Carbon monoxide may inhibit the proliferation of effector T cells, thus reducing the immune response and prevent autoimmunity and/or graft reaction [73,74]. Dietary antioxidants, in particular polyphenols, has been shown to increase HO-1 expression in different in vitro systems [3,75,76] and the potential use of this natural substances to regulate immune response should be carefully addressed.

### Antioxidants, Cancer And Neurodegenerative Disorders

It is well known that the dietary consumption of fruits, vegetables, herbs, or their phytochemical constituents aid in cancer prevention [77-79]. It is believed that the antioxidant properties of such foods protect cells from ROS-mediated DNA damage that can result in mutation and subsequent carcinogenesis. ROS-induced DNA damage can take many forms, ranging from specifically oxidized purine and pyrimidine bases, to DNA lesions such as strand breaks, sister chromatid exchanges (SCEs), and the formation of micronuclei [80]. However, the equation "antioxidant = benefit" is not always true. In vivo experiments demonstrated that retinol increases both the humoral and the cell-mediated immune response and could enhance immune surveillance against tumorigenesis [81-83]. Retinol may influence the immune response by quenching free radicals, which could lower the level of immunosuppressing lipid peroxides, alter arachidonic acid metabolism, etc. [82,84]. In the last few years many studies have been conducted to investigate the effects of vitamins on disease prevention. The first results have been encouraging and a wide number of people are taking antioxidant supplements with the aim to improve their health. These studies, initially, have shown that a high consumption of fruit and vegetables decreases risks of lung cancer in healthy individuals and a combination of β-carotene, vitamin E and selenium reduced stomach cancer mortality in China [85,86]. Conversely, supplemental β-carotene alone or in combination with retinol or vitamin E did not have any effect on cancer risk, or increased the development of lung cancer in smokers [87,88]. In the light of these first contrasting result, and also as a consequence of the wide antioxidant consumption in the general population, various systematic reviews to estimate the association between antioxidant use and disease prevention, in particular for primary cancer incidence and mortality, have been issued. These reviews share the opinion that antioxidant supplementation per se does not prevent cancer. On the contrary, some antioxidant elements seem to be harmful fro health. Recent studies have confirmed the relationship between beta-carotene and an increased incidence of cancer among smokers, but not among non-smokers. Moreover, beta carotene supplementation is associated with increased cancer-related mortality [89]. Vitamin E treatment also appears to be associated with a slightly increased incidence of lung cancer [90]. Other studies report that combination of vitamin A and other antioxidants, significantly increases mortality related to neoplastic diseases [91]. According to these studies, selenium would be the only element displaying beneficial effects, as it has been shown that it reduces total cancer incidence, an apparently sex-related effect, as it is predominant among males, rather than in females [89].

The reason why β-carotene may exert dual activity, namely antioxidant or pro-carcinogenic has been debated for quite a long time. The first hypothesis is that at high concentrations, β-carotene stimulates free radical production, whereas at lower concentrations β-carotene exerts antioxidant activity [90,91]. Furthermore, in the presence of cigarette smoke-derived free radicals β-carotene is cleaved into many derivatives which are very unstable and may trigger further oxidation [92-95]. A recent corollary to this theory is the evidence that β-carotene, either alone or in combination with cigarette smoke condensate, repressed HO-1 expression both in rat fibroblasts and human lung cancer cells [96]. The reduced expression of HO-1 accounted for a reduced production of CO and BR both of which have a marked antiproliferative effects [96-100]. Vitamin E has also been shown to act at the immune system level; in fact, supplementation with this vitamin can increase production of antibodies and enhance cell-mediated immunity in both experimental animals and in humans [101].

Neurodegenerative diseases, such as Parkinson's disease (PD), Alzheimer's disease (AD), and amyotrophic lateral sclerosis (ALS), as well as multiple slerosis (MS), are triggered, at least in part, by oxidative and nitrosative stress and also sustained by inflammatory cytokine production [11,70,102-104]. Similarly, autoimmunity mainly contributes to the pathogenesis of MS, characterized by central and peripheral loss of nerve myelin [105,106]. Although the specific sources of the damaging ROS and the affected target structures differ between the neuronal pathologies, the following general features can be defined. Increased levels of oxidation-altered metabolites are found in post-mortem tissues in many of the neurodegenerative diseases listed above [107-113]. An oxidative stress response and compensatory defense reactions can be seen in the affected neural cells; further, disturbances of the mitochondrial metabolism are observed, which may account for an increased leakage of ROS originating from the respiratory chain [11,70,104,114]. However, in addition to the direct induction of oxidative stress, metabolic disorders underlying every single disease can also indirectly generate an oxidative microenvironment, for example via the induction of a local immune response [115,116]. On this basis, antioxidant and antinflammatory drugs, such as polyphenols and non-steroidal antinflammatory drugs (NSAIDs), have been proposed in the treatment of different neurodegenerative diseases [117-119]. However, both polyphenols and NSAIDs gave rise to some problems when used in clinical setting. Due to their scarce bioavailability, only a negligible amount of polyphenols reaches brain tissue and the concentrations achieved are much lower than those efficacious in vitro [3]. As far as NSAIDs, *ad hoc *designed clinical trials with a large number of patients, clearly demonstrated that these drugs do not have any significant effect in slowing cognitive decline in patients suffering from mild-to-moderate AD [120,121]. Similar disappointing results have been obtained in the treatment of ALS, a systemic motor neuron disease that affects corticospinal and corticobulbar tracts, ventral horn motor neurons and motor cranial nerve nuclei [122,123]. Approximately 10% of cases are familial and have been linked to point mutation in the gene encoding for Cu/Zn superoxide dismutase (SOD) [124]. Mice transgenic for mutated SOD1 develop symptoms and pathologies similar to those in human ALS. Mutant SOD1 toxicity is mediated by damage to mitochondria in motor neurons, and this may trigger the functional decline of motor neurons and the onset of ALS in mice [125]. Unfortunately, although the role played by free radical to the pathogenesis of ALS has been demonstrated, antioxidants did not have any effect to prevent or slow down its progression. Desnuelle et al., clearly demonstrated that alpha-tocopherol, given together with riluzole, did not affect the survival and motor functions in ALS patients respect to the group treated with riluzole alone [126]. Novel compound, such as AEOL-10150 (Aeolus), structurally related to the SOD catalytic site, is under phase I clinical investigation, but further clinical trials will be necessary to evaluate the real efficacy of this compound for the therapy of ALS [127,128].

## Conclusion

The field of antioxidants is moving rapidly. About 20 years ago the hypothesis that diet might have a substantial influence on the development of some pathologies, such as cancer, has been raised by many scientists. In this light, during the last decade, efforts have been made to analyze the effects of plant food and synthetic antioxidants on the development and prevention of chronic diseases. Nowadays, antioxidants are used on a large scale to try to obtain and preserve optimal health. While there is no doubt that the correct balance between endogenous and exogenous antioxidant capacity is essential to life, the curative power of antioxidants has often been overestimated. In fact, according to the popular idea "if one is good two is better", antioxidants are taken in excess too often and the risk to originate diseases instead of preventing them is quite high. It is noteworthy to underlie that as for all drugs, antioxidants may give important side effects if not correctly used or in combination with other drugs. Vitamin A, E and β-carotene for instance, have been shown to have pro-oxidant effects at higher doses or under certain conditions [39].

Another point of criticism is the possibility to take experimental results "from the bench to the bedside". In fact, although the promising results obtained by in vitro experiments, the use of antioxidants in the treatment of human disease states has not been as successful as might have been envisaged due to intrinsic pharmacokinetic or pharmacodynamic limitations.

In addition, conclusions on beneficial effect of antioxidant are often drawn from studies conducted with synthetic antioxidant supplement, whereas fruits and vegetable are a complex mixture of antioxidant, as well as other potentially beneficial micronutrients and macronutrients, which may, thus, work with different kinetics and dynamics [89].

In conclusion, the correct use of antioxidants may be useful to prevent free radical-related disorders. However, the repair of existing critical structural damage may be beyond the possibilities of antioxidants and therefore they may not be considered to be useful in therapeutic clinical applications, where their limits and eventual side effects must be better understood.

## Competing interests

The authors declare that they have no competing interests.

## Authors' contributions

DB & GC wrote the manuscript. MRS & GDB edited the manuscript and did the work on references. CM contributed to the work with update of data on effects of antioxidants. PB, SP & LL contributed to the literature as recipients of a grant for the study of the effects of antioxidants in cancer cells and in neurodegeneration models. RB conceived of the study, and participated in its design and coordination. All authors read and approved the final manuscript.
